# The excessive generalization of fear affected by perceptual bias in experimental pain individuals: Evidence from an event‐related potential study

**DOI:** 10.1002/brb3.3050

**Published:** 2023-05-03

**Authors:** Xiaomin Huang, Jiali Chen, Xianglong Wang, Xuefei Zhang, Junqin Ma, Sishi Liu, Xinli Liu, Qiling Ou, Wenwei Tan, Wen Wu

**Affiliations:** ^1^ Department of Rehabilitation Medicine Zhujiang Hospital, Southern Medical University Guangzhou Guangdong China; ^2^ School of Rehabilitation Medicine Southern Medical University Guangzhou PR China

**Keywords:** attention, event‐related potentials, experimental pain, fear generalization, perception

## Abstract

**Introduction:**

Excessiv generalization of fear contributes to the development and maintenance of pain. Prior research has demonstrated the importance of perception in fear generalization and found that individuals in painful conditions exhibited perceptual bias. However, the extent to which perceptual bias in pain affects the generalization of pain‐related fear and its underlying neural activity remains unclear.

**Methods:**

Here, we tested whether perceptual bias in experimental pain individuals led to the overgeneralization of pain‐related fear by recording behavioral and neural responses. To this end, we established an experimental pain model by spraying capsaicin on the surface of the seventh cervical vertebra of the participant. A total of 23 experimental pain participants and 23 matched nonpain controls learned fear conditioning and then completed the fear generalization paradigm combined with the perceptual categorization task.

**Results:**

We found that the novel and safety cues were more likely to be identified as threat cues in the experimental group, resulting in higher US expectancy ratings compared to the control group. The event‐related potential results showed that the experimental group exhibited earlier N1 latency and smaller P1 and late positive potential amplitudes than those in the control group.

**Conclusion:**

Our findings suggest that the experimental pain individuals exhibited an excessive generalization of fear affected by perceptual bias and reduced their attentional allocation to pain‐related fear stimuli.

## Graphical abstract

The current study established an experimental pain model, using the event‐related potential to investigate the neural mechanism of fear generalization. Our results suggest that the perceptual bias in experimental pain contributes to the overgeneralization of pain‐related fear, which may lead to reduced attention to the pain‐related stimulus.

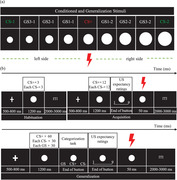

## 1 INTRODUCTION

Pain is a medical problem of global concern, leading to long‐term disability (Tsang et al., 2008). Based on previous research (Seminowicz & Moayedi, 2017), the transition from acute to chronic pain is maladaptive and results in the development of chronic clinical conditions affecting multiple components, such as sensory, emotional, cognitive and behavioral elements.

Growing evidence suggests that pain‐related fear plays a key role in the transition from acute to chronic pain (Meulders et al., 2017). According to the fear‐avoidance model, one neutral stimulus (conditioned stimulus, CS+) paired with a painful stimulus (unconditioned stimulus, US) would become a threat stimulus and elicit fear responses, while another stimulus (CS–) that was not paired with a US would be perceived as a safety one. Such fear response would not be restricted to the initial threat stimulus, but can also spread to novel stimuli (generalization stimuli, GS) based on their similarity to the CS+. This process is called fear generalization (Geschwind et al., 2015). Generalization of pain‐related fear leads to behavioral avoidance, which can be adaptive (Dunsmoor & Paz, 2015; Honig & Urcuioli, 1981). However, overgeneralization, such as excessive pain‐related fear of a wide spectrum of situations or stimuli, may lead to dysfunctional avoidance behaviors and ultimately to severe pain disability (Vlaeyen, 2015).

Previous researches investigated the fear generalization in patients with chronic pain, such as chronic hand pain (Meulders et al., 2014) and fibromyalgia patients (Meulders, Harvie et al., 2015). They found that the chronic pain population tended to overgeneralize pain‐related fear, which might lead to severe pain disability. However, due to the mismatch and confounding factors in patients with clinical acute pain (Mao, 2009), little is known about the mechanisms of fear generalization in the context of acute pain. Recently, human experimental pain models have been widely applied to understand the physiological and psychological aspects of pain. Capsaicin is commonly used to induce experimental pain and has been proven to be a safe, noninvasive approach for producing stable, persistent and reproducible painful conditions to evaluate pain mechanisms (Price et al., 2018; Shenoy et al., 2011; Silberberg et al., 2015).

EEG is sensitive to fast and transient cortical processes, allowing the exploration of temporal neural activity at different sensory and cognitive levels. Fear conditioning can modulate early and late components such as P1, N1, P2, and late positive potential (LPP). The P1 component, peak between 100 and 130 ms (Luck, 2014), was used to explore the dynamic of early attentional control (Clark & Hillyard, 1996). Evidence from several studies showed that threatening stimuli elicited enhanced P1 amplitudes (Gupta et al., 2019; Krusemark & Li, 2011; Stefanou et al., 2019). The subsequent component is N1 (peak at 100–150 ms poststimulus), which indicates the extent to which fearful stimuli allocated attentional resources (Flor et al., 2002; Rothemund et al., 2012). The CS was thought to have no effect on N1 amplitudes, but profoundly reduced the latencies of N1 (Scaife et al., 2006). Another component used to assess the brain activity to fearful stimuli is P2, which is consistently associated with anterior cingulate cortex activity (Garcia‐Larrea et al., 2003). The P2 component was found to be important in pain modulation during the emotional process (Petrovic & Ingvar, 2002; Price, 2000). A previous study has revealed increased amplitudes in the P2 component for unexpected fearful versus neutral faces (Yang et al., 2010). LPP, beginning at 300 ms after the onset of stimuli, was widely used to explore attentional and emotional processing (Pavlov & Kotchoubey, 2019). Numerous studies have shown enhanced LPP amplitudes in threat cues compared to safety cues (Bacigalupo & Luck, 2018; Panitz et al., 2015; Panitz et al., 2018; Pastor et al., 2015). LPP was a useful tool for examining individual differences in fear conditioning. However, the LPP amplitude showed no difference among generalized stimuli, thus suggesting that LPP was insensitive to fear generalization (Bauer et al., 2020; Nelson et al., 2015). Based on the research above, we were interested in assessing the neural mechanism of fear generalization in individuals with pain by analyzing the average ERP response across P1, N1, P2, and LPP.

Zaman et al. (2015) noted that individuals with chronic pain showed an impairment of perceptual discrimination, even though a growing body of studies has applied perceptually similar stimuli to explore pain‐related fear generalization (Meulders, Harvie et al., 2015; Meulders et al., 2013; Meulders & Vlaeyen, 2013) and showed an overgeneralization of fear in pain states (Meulders et al., 2014; Meulders, Jans et al., 2015). However, whether such impairment would also exist in acute pain individuals and whether it would lead to an overgeneralization of pain‐related fear still remains unclear. To fill this gap, we applied a generalization protocol developed by Struyf et al. (2017), which combined the paradigm of fear generalization and a categorization task to evaluate generalization behavior and stimulus perception simultaneously. In this experiment, continuously sized circles served as CSs (Lissek et al., 2008). The middle‐size circle was paired with an electrical stimulus (den Hollander et al., 2015; Glogan et al., 2019; Karos et al., 2015; Vandael et al., 2019) to elicit fear responses. During the generalization phase, one stimulus (either CS or GS) was presented in each trial and EEG was recorded. Then, a categorization task appeared, requiring participants to categorize the current stimulus as CS or GS. After categorizing the stimulus, participants were asked to rate the US risk expectancy. A higher US expectancy ratings reveals a stronger fear response. Based on previous research, we hypothesized that compared to the control group, experimental pain individuals may exhibit perceptual bias which contributed to the overgeneralization of pain‐related fear. Second, the ERP component would differentiate CS from GS and show a generalization gradient. Moreover, since the prior study had demonstrated that individuals with experimental pain could not pay as much attention to cognitive tasks as pain‐free controls (Wang et al., 2020), the third hypothesis was that the amplitudes of the ERP components in the experimental group would be smaller than that in the control group.

## 2 MATERIAL AND METHODS

### 2.1 Participants

A total of 46 healthy individuals (age 18–35, 28 females, mean age = 23.24 ± 2.76) were recruited from the Southern Medical University. One of the females was left‐handed, while the others were right‐handed. Participants had a normal or corrected‐to‐normal vision. The exclusion criteria included any forms of pain, current/past neurological disease or psychiatric disorders and/or current medication use. Twenty‐three participants were randomly assigned to the experiment group (experimental pain, EP, 14 females) and the other 23 were assigned to the control group (nonpain, NP, 13 females). The 14‐item Hamilton Anxiety Rating Scale (HAMA) and the Hamilton Depression Rating Scale‐24 item (HAMD‐24) were assessed before the experiment to evaluate depressive and anxiety symptoms between the EP and NP groups. To test the cognitive attitudes toward pain, we also applied the Fear of Pain Questionnaire‐III‐Chinese (FPQ‐III‐C) to the EP and NP groups (McNeil & Rainwater, 1998). The FPQ‐III‐C is used to assess fear of different causes of pain including 30 items (McNeil & Rainwater, 1998). Table 1 shows the demographic and questionnaire data. No significant group differences emerged. After being fully familiar with the experiment process, all participants gave written informed consent and received 80RMB for their participation. The study was approved by the Ethics Committee of the Zhujiang Hospital of Southern Medical University.

**TABLE 1:** Demographic and questionnaire scores for the experimental pain group and control group (*M* ± *SD*).

	EP	NP	χ^2^/*t* value	*p* Value
Sex (male/female)	9/14	10/13	0.090	.765
Age	23.348 ± 3.298	23.130 ± 2.160	–0.264	.502
BMI	21.843 ± 2.829	21.889 ± 5.470	0.036	.372
HAMA‐14	1.957 ± 1.461	1.304 ± 1.845	–1.329	.724
HAMD‐24	2.522 ± 1.275	1.696 ± 1.579	–1.952	.103
Intensity of US	0.465 ± 0.157	0.442 ± 0.149	–0.520	.489
FPQ‐III‐C	3.025±0.647	3.029±0.567	0.024	.981

Abbreviations: EP, experimental pain group; NP, control group; HAMA‐14, the 14‐item Hamilton Anxiety Rating Scale; HAMD‐24, the Hamilton Depression Rating Scale‐24 item; FPQ‐III‐C, Fear of Pain Questionnaire‐III‐Chinese.

### 2.2 Experimental pain model

To explore fear generalization in acute pain conditions, we established an experimental pain model induced by capsaicin in the EP group throughout the experiment. For the model, capsaicin powder (Sigma) was dissolved in 50% ethanol in water at a concentration of 0.1%, sprayed on the surface of the seventh cervical vertebra and covered with plastic wrap. The NP group was sprayed with pure water and covered with plastic wrap (Wang et al., 2020). The visual analog scale (VAS) was used to assess pain intensity with a target of 4, indicating that the sensation was starting to become painful. During the experiment, we continuously assessed the pain intensity between blocks. Once the participant's pain perception dropped below 4/10 on the VAS, capsaicin was applied to maintain the pain perception score above 4/10.

### 2.3 Conditioned and unconditioned stimuli

**FIGURE 1:**
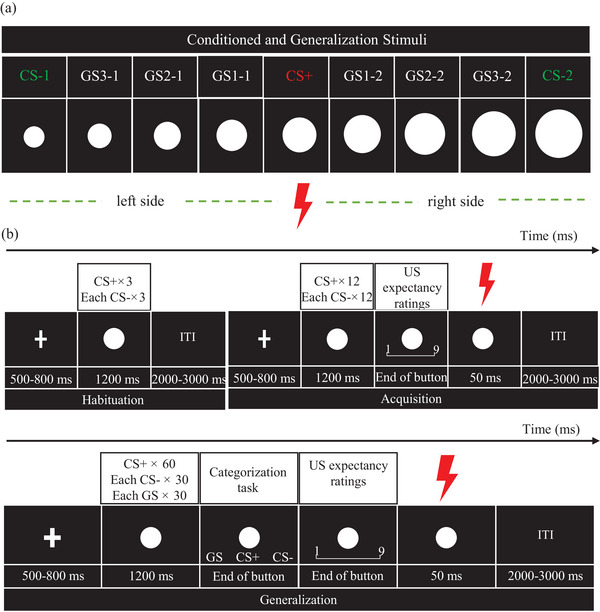
Experimental overview. (a) Overview of the conditioned and generalization stimulus. (b) Both the experiment and control group underwent the habituation, acquisition, and generalization phases. During the habituation phase, CS+ or CS– was presented for 1200 ms following a fixation cross. In the acquisition phase, US expectancy ratings appeared after the presentation of the stimulus, then 9 out of the 12 CS+ were paired with electrical shock as the US. In the generalization phase, after displaying the CSs or GSs, a categorization task appeared that required participants to identify the current stimulus as either CS+, CS– or as a novel stimulus, then followed by US expectancy ratings. CS = conditioned stimulus, US = unconditioned stimulus, GS = generalization stimulus.

According to previous studies, nine white circles of gradually increasing diameters (from 5.08 to 11.18 cm, in steps of 0.762 cm; see Figure 1A) were presented on a black background (Lissek et al., 2008). The medium‐sized circle (8.13 cm) served as CS+, paired with US (75% reinforcement), while the smallest (5.08 cm, CS‐1) and the largest one (11.18 cm, CS‐2) served as CS– presented alone (Cha et al., 2014). The remaining six circles on two sides of the CS+ served as GSs (Circle2, GS1‐1; Circle 8, GS1‐2; Circle3, GS2‐1; Circle 7, GS2‐2; Circle4, GS3‐1; Circle 6, GS3‐2).

An electrical stimulus served as an unconditioned painful stimulus (pain‐US) using the BIOPAC stimulator module (STM200 BIOPAC Systems Inc., Santa Barbra, CA). The US was attached to a pair of electrodes (9 mm diameter, 30 mm distance between electrodes) filled with K‐Y gel. The electrodes were placed on the tibial surface of the leg (the stimulated side was balanced across participants) about 10 cm above the malleolus (van de Donk et al., 2019) to avoid muscle contractions. The intensity of US was individually calibrated using a series of ascending electrical stimuli with a step of 0.02 V and an interstimulus interval ranging from 7 to 15 s (Carlino et al., 2010). After the presentation of each stimulus, participants scored subjective perception/pain intensity using the 11‐point Likert scale. Here, 0 = “You don't feel anything at all,” 1 = “You feel something, but it's not painful, it's merely a sensation,” 2 = “The sensation starts to become aversive, but it's still not painful” and 10 = “This is the worst pain you can imagine.” The target intensity for the US was “significantly painful and demands some efforts to tolerate,” which was roughly equivalent to a score of 8 on this calibration scale (Meulders, Harvie et al., 2015) (Table 1). Participants were instructed to notify the experimenter at any time when they were unable to withstand a higher intensity stimulus, or when they wanted to reduce the intensity to a lower level. Following this procedure, participants were asked if they agreed to repeatedly receive the stimuli of maximal chosen intensity during the experiments (Vandael et al., 2019).

### 2.4 Procedure

The participant was seated in a sound‐attenuated, dimly lit room. The experiment was programmed using E‐prime3.0 (Psychology Software Tools Inc., Pittsburgh, USA) on a 21‐inch computer monitor with a resolution of 1920 × 1080 pixels and a refresh rate of 60 Hz, placed approximately 60 cm in front of participants.

There were three consecutive phases: habituation, acquisition and generalization (see Figure 1B), in which trials were carried out in quasi‐random order such that the same stimuli occurred no more than twice in a row. During the habituation phase, the CS‐1, CS‐2 and CS+ were presented 3 times each. Trials started with a fixation cross for 500–800 ms, followed by a CS presented for 1200 ms, with intertrial intervals (ITI) ranging from 1000 to 3000 ms. In the acquisition phase, 12 CS+, 12 CS‐1, and 12 CS‐2 each presented for 1200 ms and were divided into three blocks. To explore the conditioned response between the EP and NP groups, after the presentation of the CS, participants were asked to rate the level of US expectancy on a 9‐point scale (1 = least likely, 5 = moderately likely, 9 = most likely) with their right hand. After answering, 9 out of the 12 CS+ were followed by the US (75% reinforcement rate, 50 ms) while the CS– was never followed by the US. The generalization phases consisted of 10 blocks. A categorization task was performed aiming to explore the role of perception in fear generalization. In each block, CS+ was presented 6 times and CS‐1, CS‐2, and GSs were presented 3 times each. After the CS or GS was presented for 1200 ms, the categorization task appeared at the bottom of the circle. The participant was required to identify whether the circle was the stimulus previously judged as 1) a novel stimulus (GS), 2) the best predictor of US (CS+) or 3) the stimulus, which did not predict US (CS–). They pressed button “J” with their left hand when they categorized the current circle as GS and pressed button “K” when they thought the current circle was CS+, while button “L” was pressed when they identified the current circle as CS–. After the categorization task, participants were asked to rate the US expectancy with their right hand. To avoid extinction, 3 of the 6 CS+ in each block were paired with US (50% reinforcement rate).

### 2.5 EEG recording and preprocessing

The scalp EEG was recorded at a sampling rate of 2048 Hz from 64 Ag/Ag‐Cl scalp electrodes mounted according to the international 10–20 system using the 64‐channel BIOSEMI Active Two system. The average value of bilateral mastoids served as an online reference. Brain activity was continuously recorded over a band‐pass range of 0.01–100 Hz, with electrode impedances kept below 20 kΩ.

The offline analysis of EEG data was processed using the EEGLAB toolbox (version 13_0_0b) (Delorme & Makeig, 2004) in Matlab (R2013b, MathWorks, Natick, MA, USA). After loading the raw data, 64 electrodes were selected and located. The EEG data were down‐sampled to 256 Hz. Additionally, the EEG signals were band‐pass filtered between 0.5 and 30 Hz and notch filtered between 49 and 51 Hz. The EEG epochs were segmented using 1200 ms time windows (prestimulus –200 ms and poststimulus 1000 ms) and the baseline was corrected using the prestimulus time interval. Epochs containing obvious artifacts were manually removed for each participant by visual inspection. Then, we used the Automatic Channel Rejection tool to identify bad channels and interpolated through the Interpolate Electrodes. The average number of interpolated channels was 2.94. Independent component analysis (ICA) was subsequently performed to remove components associated with eye movements and blinking. Epochs with activity exceeding ±100 μV were rejected using a semi‐automated procedure. Finally, clean EEG data epochs were merged and averaged for each condition at the group level to obtain the grand average ERP waveform.

### 2.6 Data analysis

In the acquisition phase, for the behavioral data, we analyzed the US expectancy ratings using a three‐way repeated‐measures analysis of variance (RMANOVA) with Stimulus (CS+ and CS–) and Block (blocks 1–3 of acquisition phase) as within‐subject factors and Group as the between‐subject factor. In the generalization phase, we first aimed to investigate whether there was perceptual bias in the EP group compared with the NP group. We converted the categorization data into the probabilities of being categorized as CS+, GS, and CS– for each test stimulus and analyzed using a two‐way RMANOVA [Stimulus (CS+, CS–, GSs) × Group] in these three categories, separately. Then, we analyzed the US expectancy ratings in two steps. In the first step, we averaged the ratings for each type of stimulus in each group across blocks 1 to 10 in the generalization phase, using RMANOVA with Stimulus and Block as within‐subject factors and Group as the between‐subject factor. We expected the EP group to have higher US expectancy ratings than the NP group, indicating an overgeneralization of pain‐related fear in the EP group. In the second step, we performed three‐way RMANOVA [Stimulus × Category (CS+ category, GS category and CS– category) × Group] to analyze the US expectancy ratings when the stimuli were categorized as CS+, GS, and CS–, respectively, to explore the group differences and the role of perception in the US expectancy ratings.

To have an adequate signal‐to‐noise ratio for the ERP analysis, we merged the data of circles on the opposite sides of CS+ into GS1, GS2, GS3, and CS–. The average number of analyzed epochs in ea condition per participant are shown in Table 2. The mean amplitudes and peak latencies of components were analyzed by selecting electrode sites for the specific time windows based on previous studies and visual inspection. For N1 and P2 components, the mean activities of 5 electrode sites (C1, C2, C3, C4, and Cz) for each stimulus type were extracted over the time windows of 100–130 ms and 160–180 ms, respectively. A three‐way RMANOVA was conducted with Stimulus (CS+, GS1‐3, and CS–) and Electrode (left, middle, and right) as within‐subject factors and Group as the between‐subject factor for the analysis of N1 and P2 components. As for P1, the amplitudes and peak latencies of the two electrode sites (PO7 and PO8) for each stimulus type were averaged over a time window of 90–115 ms. In addition, the mean amplitude and peak latency of LPP were extracted at electrode sites (CP3, CP4, CP5, and CP6) in a time window of 400–800 ms. A 5 × 2 × 2 RMANOVA was performed with Stimulus (CS+, GS1‐3, CS–) and Electrode (left and right) as within‐subject factors and Group as the between‐subject factor for the analysis of P1 and LPP components. SPSS 22 (IBM Corp, Armonk, NY, USA) was applied for all data analysis. The Greenhouse–Geisser correction was conducted to correct *p* values and Bonferroni correction was used for multiple post hoc testing.

**TABLE 2:** The average number of analyzed epochs in each condition per participant (*M* ± *SD*).

Group	Stimulus				
	CS+	GS1	GS2	GS3	CS–
EP	51.17 ± 5.441	51.78 ± 4.602	51.87 ± 4.948	50.09 ± 5.418	49.83 ± 6.125
NP	50.39 ± 5.574	51.35 ± 5.424	50.87 ± 6.566	48.52 ± 6.775	46.96 ± 7.553

Abbreviations: EP, experimental pain group; NP, control group; CS, conditioned stimulus; GS, generalization stimulus.

## 3 RESULTS

### 3.1 Behavioral results

**FIGURE 2:**
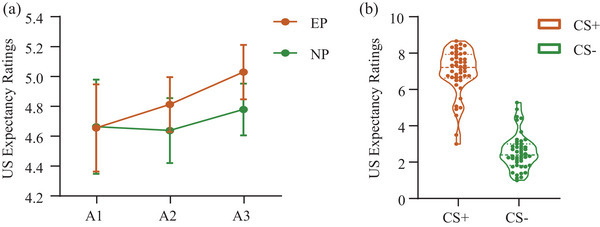
Participants’ US expectancy ratings in the acquisition phases. (a) Mean US expectancy rating judgments for the EP and NP groups separately during both acquisition blocks (A1–3). (b) Violin plots for the US expectancy ratings for the CS+ and the CS– during the acquisition phase. CS = conditioned stimulus, GS = generalization stimulus, EP = experimental group, NP = control group. Error bars represent standard errors. ***p ≤ .001, **p ≤ .01, *p ≤ .05.

In the acquisition phase, the US expectancy ratings were significantly modulated by the main effect of Stimulus [*F* (1, 44) = 369.796, *p* < .001, *n* ^2^ *p* = 0.894]. This main effect was qualified by a significant interaction with Block [*F* (2, 88) = 38.637, *p* < .001, *n* ^2^ *p* = 0.468], such that the responses to CS+ were higher than those to CS– in each block (all *p* values < .001). The US expectancy ratings to CS+ were significantly lower in block 1 than those in blocks 2 and 3, while the responses to CS– were significantly higher in block 1 than those in blocks 2 and 3 (all *p* values < .001). We found no statistically significant between‐group difference in the acquisition phase (Figure 2A). The results in the acquisition phase indicated successful learning of the contingency between CS and US (Figure 2B)

**FIGURE 3:**
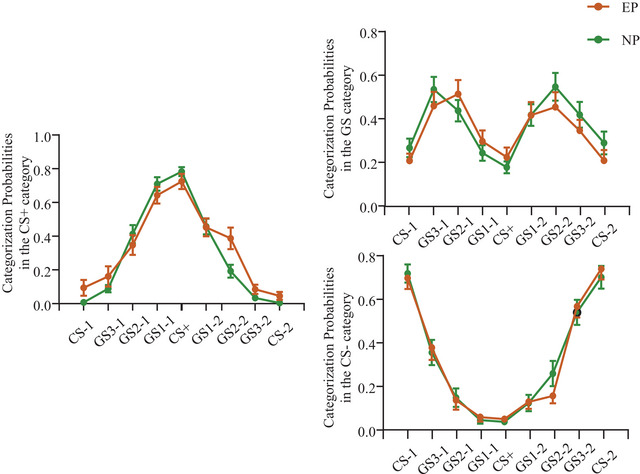
Categorization probabilities in the generalization phase. Categorization probabilities across stimulus with trials on which the stimulus was categorized as CS+ (left), GS (upper right), and CS– (lower right). CS = conditioned stimulus, GS = generalization stimulus, EP = experimental group, NP = control group. Error bars represent standard errors. ***p ≤ .001, **p ≤ .01, *p ≤ .05.

In the generalization phase, we were interested in the probabilities of being categorized as CS+, GS, and CS– for each test stimulus in the categorization task. When the test stimulus was judged as CS+, the two‐way RMANOVA reached a significant main effect of Stimulus [*F* (2.772, 121.953) = 110.675, *p* < .001, *n* ^2^ *p* = 0.716]. More importantly, we observed a Stimulus × Group interaction [*F* (2.772, 121.953) = 2.772, *p* = .049, *n* ^2^ *p* = 0.059]. The simple effects demonstrated that the EP group showed a significantly higher probability to categorize GS2‐2 as CS+ (*p* = .012) and marginally higher probabilities to misidentify the CS‐1, CS‐2, and GS3‐2 as CS+ (*p* ≤ .08) compared with the NP group. In the condition that the test stimulus was judged as GS or CS–, we only found significant main effects of Stimulus (all *p* values < .001), but no statistically significant difference involving Group and Block (Figure 3).

**FIGURE 4:**
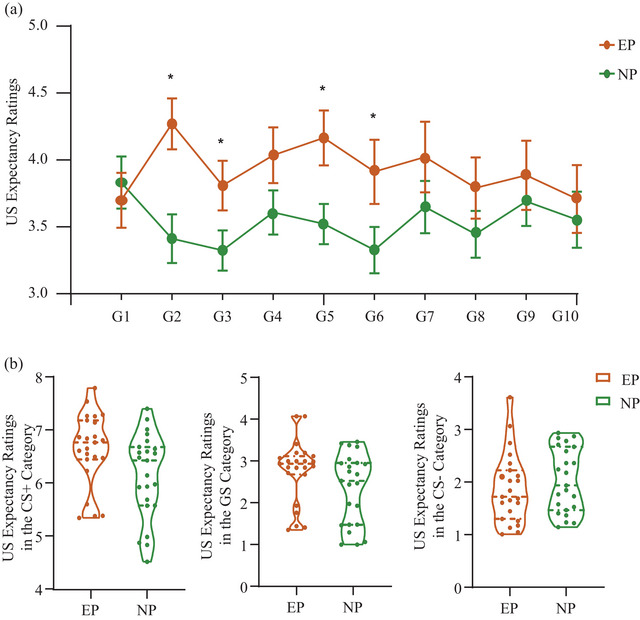
Subjective US expectancy ratings in the generalization phase. (a) Mean US expectancy rating judgments for the CS and the GSs for the EP and NP groups separately during both generalization blocks (G1–10). (b) US expectancy ratings across stimulus with trials on which the stimulus was categorized as CS+ (left), GS (middle), and CS– (right). CS = conditioned stimulus, GS = generalization stimulus, EP = experimental group, NP = control group. Error bars represent standard errors. ***p ≤ .001, **p ≤ .01, *p ≤ .05.

As revealed by the three‐way RMANOVA, the US expectancy ratings were modulated by the main effect of Stimulus [*F* (2.508, 110.352) = 157.432, *p* < .001*, n* ^2^ *p* = 0.782]. These ratings were also significantly modulated by the interaction between Block and Stimulus [*F* (17.402, 765.700) = 3.271, *p* < .001*, n* ^2^ *p* = 0.069]. The US expectancy ratings were significantly higher to CS+ and GSs than those to CS– (all *p* values < .05), indicating successful fear generalization to GSs. The Block × Group interaction also reached significance in the analysis of US expectancy ratings [*F* (4.233, 186.272) = 2.706, *p* = .029*, n* ^2^ *p* = 0.058]. The follow‐up contrast demonstrated that the EP group showed significantly higher responses in blocks 2, 3, 5, and 6 than those in the NP group (all *ps* < 0.05). These results reflected the overgeneralization of pain‐related fear in the EP group (Figure 4A).

As a follow‐up to the perceptual categorization effect, we analyzed the US expectancy ratings when the test stimulus was categorized as CS+, GS, and CS–, performing a Stimulus × Category × Group RMANOVA (Figure 4B). The ratings were modulated by the main effect of Stimulus [*F* (4.166, 183.321) = 74.972, *p* < .001, *n* ^2^ *p* = 0.630]. The ratings were also modulated by the main effect of Category [*F* (2, 88) = 1.960, *p* < .001*, n* ^2^ *p* = 0.915]. More importantly, we also found a significant three‐factor interaction between Stimulus, Category, and Group [*F* (9.238, 406.472) = 2.042, *p* = .032*, n* ^2^ *p* = 0.044]. Hence, we calculated post hoc two‐way RMANOVA with these three factors separately.

We first performed two‐way ANOVA on Stimulus and Category in the EP and NP groups, separately. In both the EP and NP group, the ratings were modulated by the Stimulus [EP, *F* (3.103, 68.274) = 35.884, *p* < .001*, n* ^2^ *p* = 0.620; NP, *F* (4.357, 95.847) = 41.204, *p* < .001*, n* ^2^ *p* = 0.652]. The ratings were also modulated by the main effect of Category [EP, *F* (2, 44) = 216.733, *p* < .001, *n* ^2^ *p* = 0.908; NP, *F* (2, 44) = 266.392, *p* < .001*, n* ^2^ *p* = 0.924]. The main effect was qualified by the interaction with Stimulus in both the EP [*F* (6.326, 139.177) = 9.363, *p* < .001*, n* ^2^ *p* = 0.299] and NP group [*F* (8.118, 178.587) = 6.595, *p* < .001*, n* ^2^ *p* = 0.231]. The post hoc test revealed that the participants showed higher US expectancy ratings in the CS+ category than those in GS and CS– categories in the two groups (all *p* values < .001). In the EP group, participants reflected higher ratings in the GS category than in the CS– category (all *p* values < .05), while no such difference was found in the NP group (*p* > .05).

Then, we performed the two‐way repeated‐ measures ANOVA between Stimulus and Group in each category. In the CS+ category, the analysis showed significant main effect of Stimulus [*F* (4.725, 207.914) = 69.364, *p* < .001*, n* ^2^ *p* = 0.612]. The ratings were also modulated by the main effect of Group [*F* (1, 44) = 9.363, *p* = .028*, n* ^2^ *p* = 0.105]. The between‐group difference showed that the US expectancy ratings in the EP group (6.642 ± 0.684) was significantly higher than those in the NP group (6.143 ± 0.800). In the GS category, a main effect of Stimulus [F (4.311, 189.700) = 31.495, *p* < .001, *n* ^2^ *p* = 0.417] was observed. More importantly, we found an interaction effect between Stimulus and Group [*F* (4.311, 189.700) = 3.130, *p* = .014*, n* ^2^ *p* = 0.066], reflecting that the US expectancy rating elicited by CS+ and GS3‐2 was higher in the EP group compared with those in the NP group (all *p* values < .05). In the CS– category, we only found the main effects of Stimulus [*F* (4.371, 192.345) = 14.177, *p* < .001*, n* ^2^ *p* = 0.244], such that ratings to CS+ and GS1‐1 were higher than those to CS‐1 and CS‐2 and ratings to CS+ were higher than those to GSs (all *p* values < .01)

Finally, we analyzed the interaction between Category and Group in each type of stimulus. The ratings were modulated by the main effect of Category (all *p* values < .001) in each type of stimulus. We also found a marginally significant main effect of Group in stimulus GS1‐1, GS2‐2, and GS3‐3 (all *p* values < .1). More importantly, the results demonstrated that ratings elicited by GS3‐1 [*F* (1.724, 75.843) = 4.433, *p* = .019*, n* ^2^ *p* = 0.092] and CS+ [*F* (1.764, 77.597) = 5.368, *p* = .009*, n* ^2^ *p* = 0.109] were modulated by the interactions between Category and Group, reflecting greater responses in the EP group compared to those in the NP group in the CS+ category (all *p* values < .05).

The behavioral results indicated that participants successfully learned the CS+ as dangerous stimulus and CS– as safety one in the acquisition phase. In the generalization phase, the EP group was more likely to judge the generalization stimulus as dangerous one. Regarding to the US expectancy ratings, the EP group showed greater response than the NP group, which indicating an overgenerlization in EP group. Participants showed higher US expectancy ratings in the CS+ category than those in GS and CS– categories. We also found greater responses in the EP group compared to those in the NP group in the CS+ category. These results suggested that perceptual bias in the EP group might have an effect on the pain‐related fear generalization.

### 3.2 ERP results

**FIGURE 5:**
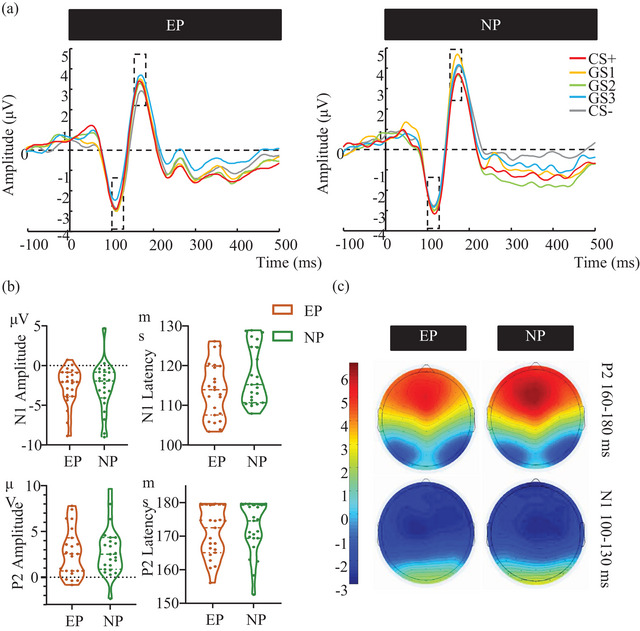
N1 and P2 components evoked by CSs and GSs in the generalization phase. (a) The grand average ERPs waveforms of the EP and NP groups. (b) The mean amplitude and latency of N1 and P2 elicited by the CS and the GSs for the EP and NP groups. (c) The topography map of the N1 and P2 waves. Note. The N1 was measured at 100–130 ms and P2 was measured at 160–180 ms. Measurements were taken from electrodes C1, C2, C3, C4, and Cz and baselined –200 to 0 ms. CS = conditioned stimulus, GS = generalization stimulus, EP = experimental group, NP = control group. Error bars represent standard errors.

The analysis of the N1 component showed no statistically significant main effects or interaction effects in averaged amplitudes (Figure 5). However, the N1 peak latency revealed a significant interaction of Stimulus × Electrode × Group [*F* (5.927, 260.794) = 3.247, *p* = .004, *n* ^2^ *p* = 0.069]. Thus, we performed post hoc two‐way RMANOVAs on the interaction between Electrodes and Group in each type of stimulus and the interaction between Stimulus and Group on each side of the electrode to further analyze the between‐group difference in N1 latency. As for the RMANOVA on Electrodes and Group, N1 latency was marginally modulated by the main effect of Group under GS2 [*F* (1, 44) = 4.666, *p* = .036, *n* ^2^ *p* = 0.096], such that the N1 latency in EP group (113.055 ± 7.956 ms) was shorter than those in the NP group (118.263 ± 8.392 ms). The N1 latency was also modulated by the interaction between Electrode and Group under GS1 [*F* (1.637, 72.029) = 3.247, *p* = .007, *n* ^2^ *p* = 0.120]. The EP group showed significantly earlier N1 peak latency in the right electrodes (EP: 112.687 ± 8.337 ms, NP: 119.395 ± 8.124 ms, *p* = .008) and a marginally shorter N1 peak latency compared with the NP group in the Cz electrode (EP: 112.772 ± 8.759 ms, NP: 118.037 ± 9.709 ms, *p* = .060). As for the RMANOVAs on Stimulus and Group, we only found a marginally significant main effect of Group in the right electrodes [*F* (1, 44) = 3.934, *p* = .054, *n* ^2^ *p* = 0.082], such that the EP group (113.553 ± 6.348ms) showed shorter N1 latency than those in the NP group (117.663 ± 7.646ms). No other main effects or interaction effects were found.

**FIGURE 6:**
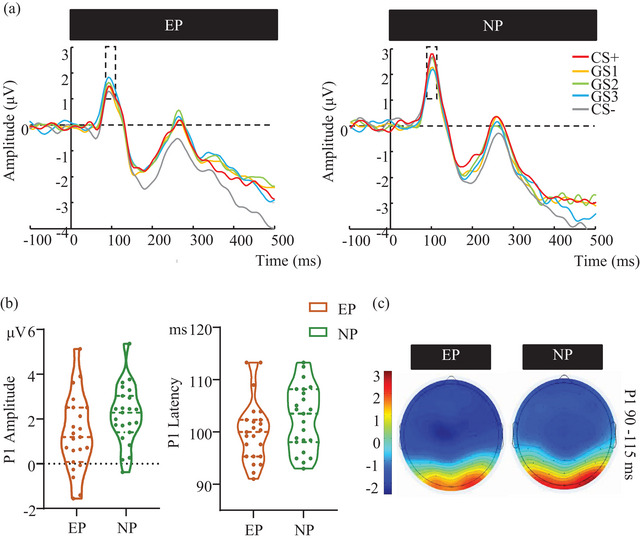
P1 components evoked by CSs and GSs in the generalization phase. (a) The grand average ERPs waveforms of the EP and NP groups. (b) The mean amplitude and latency of P1 elicited by the CS and the GSs for the EP and NP groups. (c) The topography map of the P1 waves. Note. The P1 was measured at 90–115 ms. Measurements were taken from electrodes PO7 and PO8 and baselined –200 to 0 ms. CS = conditioned stimulus, GS = generalization stimulus, EP = experimental group, NP = control group. Error bars represent standard errors. ***p ≤ .001, **p ≤ .01, *p ≤ .05.

For P1 amplitude, a significant main effect of Group [*F* (1, 44) = 4.226, *p* = .046, *n* ^2^ *p* = 0.088] was found. Crucially, this main effect was qualified by the interaction with Stimulus [*F* (4, 176) = 3.966, *p* = .043, *n* ^2^ *p* = 0.128]. The follow‐up contrast demonstrated that the EP group showed smaller amplitudes elicited by CS+, GS2, and CS– compared to the NP group (all *p* < .05). We also found a significant Electrode main effect [*F* (1, 44) = 6.268, *p* = .016, *n* ^2^ *p* = 0.125], reflecting that the P1 amplitude in PO8 (2.196 ± 1.922 μV) was more positive than that in PO7 (1.394 ± 1.805 μV). No other main effects or interactions were found. As for the peak latency, neither interaction nor the main effect was observed (Figure 6).

Regarding P2 amplitude, the main effect of Stimulus was significant [*F* (4, 176) = 4.216, *p* = .003, *n* ^2^ *p* = 0.087]. The P2 amplitude was also modulated by the main effect of Electrode [*F* (2, 88) = 25,622, *p* < .001, *n* ^2^ *p* = 0.368], reflecting that the P2 amplitude was most positive at Cz. More importantly, a significant interaction of Stimulus × Group was found [*F* (4, 176) = 3,455, *p* = .010, *n* ^2^ *p* = 0.073]. Bonferroni‐corrected pairwise comparisons yielded significantly greater P2 amplitude elicited by GS1 than GS2 (*p* = .024) in the NP group, while the EP group showed a more positive P2 amplitude elicited by GS3 compared with that elicited by CS– (*p* = .034). We found no between‐group difference in P2 amplitude. In addition, the P2 peak latency revealed no significant main effects or interactions (Figure 5).

**FIGURE 7:**
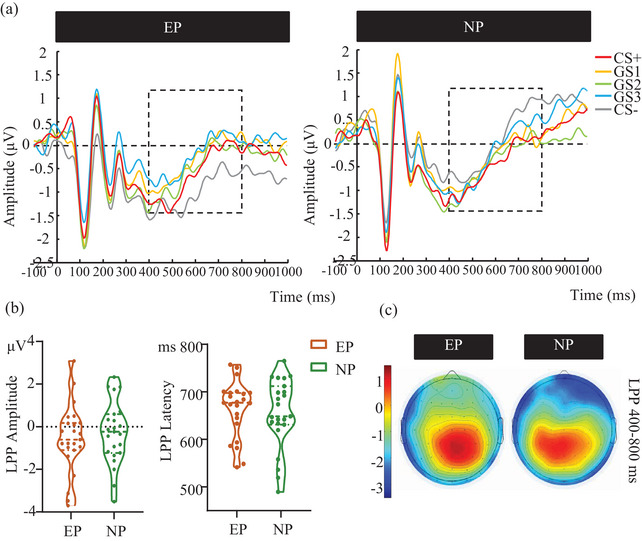
LPP components evoked by CSs and GSs in the generalization phase. (a) The grand average ERPs waveforms of the EP and NP groups. (b) The mean amplitude and latency of LPP elicited by the CS and the GSs for the EP and NP groups. (c) The topography map of the LPP waves. Note. The LPP was measured at 400–800 ms. Measurements were taken from electrodes CP3, CP4, CP5, and CP6 and baselined –200 to 0 ms. CS = conditioned stimulus, GS = generalization stimulus, EP = experimental group, NP = control group.

Analysis of LPP amplitudes (Figure 7) revealed a significant interaction between Stimulus and Group [*F* (4, 176) = 2.437, *p* = .049, *n* ^2^ *p* = 0.052]. Follow‐up simple effects tests confirmed that the LPP amplitude elicited by CS– was greater in the NP group (–0.021 ± 1.527 μV) than in the EP group (–1.024 ± 1.763 μV, *p* = .045). In addition, an Electrode‐by‐Group interaction was also shown to be significant [*F* (1, 44) = 5.924, *p* = .019, *n* ^2^ *p* = 0.119]. Post hoc contrasts confirmed that in the EP group, the LPP amplitude was more positive in the right electrode (–0.059 ± 2.268 μV) than in the left electrode (–1.110 ± 1.329 μV, *p* = .008). Besides, a between‐group difference showed that the LPP amplitude was more positive in the NP group (–0.177 ± 1.601 μV) compared with the EP group (–1.109 ± 1.329 μV, *p* = .037) in the left electrode. No other main effects or interaction effects were found. As for peak latency of the LPP component, a statistically significant difference of the Electrode [*F* (1, 44) = 14.606, *p* < .001, *n* ^2^ *p* = 0.249] reflected that the LPP peak latency of the right electrode (638.791± 79.109 ms) was earlier than that of the left electrode (680.707 ± 72.387 ms). We did not find any other main effects or interaction effects for the LPP latency.

For the ERP results, we found that the EP group showed shorter N1 latency as well as smaller P1 and LPP amplitudes than the NP group.

## 4 DISCUSSION

In the current study, we examined the role of perception in the generalization of pain‐related fear as well as the underlying neural mechanisms in a painful state. The results demonstrated that indivudual in painful condition showed perceptual bias which influenced the overgeneralization of pain‐related fear and they showed a different pattern of psychobiological processes.

The stronger fear responses in the EP group were consistent with previous research studied in fibromyalgia and chronic hand pain patients, which showed a greater fear response compared to healthy individuals (Meulders et al., 2014; Meulders, Jans et al., 2015). Growing studies provided a perceptual account for the observed overgeneralization. Hence, we combined the fear generalization paradigm with a categorization task to explore the impact of perceptual bias in the fear generalization in experimental pain. Previous study found that pain interferes with cognitive tasks via attentional disruption (Eccleston & Crombez, 1999). The categorization task can be viewed as a cognitive task, which was disturbed by the experimental pain condition in our study. This impact of pain might lead to a perceptual bias in painful sufferers. In Vriends’ (2011) study, they applied CS+ and CS– to explore the fear acquisition and extinction in individuals with state anxiety and found enhanced responses to the CS+ as well as the CS– in an anxious state. Vriends et al. found that after the fear learning in the acquisition, individuals may be prepared psychologically and transfer the emotional regulation strategies to the subsequent stage of conditioned fear. Thus, the higher ratings in the GS category than the CS– category in the EP group found in our study might be that individuals in painful conditions were induced a more alert psychological preparation after the stage of acquisition and applied the adjustment strategy of fear emotion to the generalization phase, resulting in a higher fear expectation for the stimulus which they categorized as a novel one. Apart from that, when the participants identified the current test stimuli in the same category, such as the GS category, the generalized fear responses still revealed differences based on perceptual similarity in both the EP and NP group. Consistent with previous studies (Struyf et al., 2017; Zaman et al., 2019), these results indicated that fear generalization is not only a byproduct of perception bias in experimental pain individuals, but may also be driven by other cognitive processes. The “safe‐than‐sorry strategy” might be considered an explanation for these findings in our study. In this case, participants elicited a stronger fear response, even when they were aware that the current stimulus is a novel one but not a conditioned fear stimulus, for the reason that a misidentification of the safe stimulus as the dangerous one is better than incorrect identification of the conditioned stimulus as a safe one (Dunsmoor & Paz, 2015; Shepard, 1987). This notion reflects that generalization is a postperceptual decision process.

The N1 component is mainly related to the orientation of attention in perceptual discrimination (Ohoyama et al., 2012; Vogel & Luck, 2000). Hence, the change in N1 latency may reflect the processing speed of attentional allocation (Vogel & Luck, 2000). Based on a prior study, the pain‐related fear is associated with hypervigilance (Leeuw et al., 2007), which suggests that the reduction in N1 latency is accounted for increasing vigilance in pain conditions, thus accelerating the speed of attention allocation toward the pain‐related fear stimulus. This result is consistent with the behavioral results supporting our conclusion that individuals in painful conditions applied a more alert adjustment strategy of fear emotion to the generalization phase.

Moreover, the P1 component was associated with the efficiency of visual stimulus detection through the allocation of top‐down attention (Desimone & Duncan, 1995; Hillyard & Anllo‐Vento, 1998; Luck et al., 1990). The decreased P1 amplitudes in the EP group were possibly related to the reduction of visual processing of pain‐related fear stimulus, which reflects an attentional avoidance in the experimental pain individuals. This finding is consistent with works on the fear‐avoidance model, suggesting that highly pain‐related fear individuals view pain as a sign of damage, which is linked to limited pain‐control coping strategies and results in avoidance of pain‐related movements and activities (Bunzli et al., 2017).

In the present study, we found that the behavioral results showed a different pattern from the ERP results, as the US expectancy ratings were significantly modulated by the type of stimulus. However, the P2 amplitude did not exhibit such modulation by the type of stimulus but even showed higher amplitude for novel stimulus compared with those for CS+. A dual‐process theory of conditioning states a dissociation between implicit and explicit processes and that conditioning can occur independently of explicit contingency awareness (Balderston & Helmstetter, 2010; Schultz & Helmstetter, 2010). In the present study, the US expectancy rating measures the explicit process for the CS‐US contingency awareness, while EEG is applied to explore the implicit processes to fear CSs (Lonsdorf et al., 2017). Behavioral and ERP results demonstrated that participants were capable of expressing contingency awareness, but did not show evidence of autonomic conditioning. Our study provides evidence for the dual‐process interpretation of conditioning, suggesting that implicit and explicit learning might simultaneously exist in fear conditioning.

The present study also showed that LPP exhibited the same ERP difference pattern as the P1 component, reflecting an enhanced amplitude in pain‐free conditions compared with painful conditions. These findings further demonstrate the difference in neural responses between pain and pain‐free conditions, consistent with previous research into experimental pain (Wang et al., 2020), supporting the conclusion that pain disrupts early and later neural potentials. Eccleston (1994) has proposed that pain demands attentional resources. Since experimentally induced pain has been found to impair aspects of attention (Bingel et al., 2007; Moore et al., 2013; Seminowicz et al., 2004; Wang et al., 2020), the reduction of LPP amplitude between the pain and pain‐free condition may be due to the impairment of allocation of attention in pain conditions.

Some limitations warrant comment. First, during the acquisition phase, the number of CS‐US trials was limited, which might not form strong enough conditioning effects. Second, to reduce the fatigue effect caused by a long experiment time, the presentation time of stimuli and intertrial interval was shortened. This parameter might result in considerable effects on ERP results. Finally, the present study explored fear generalization in a control experimental setting. Hence, the conclusion in this experiment could not generally apply to patients suffering from clinical acute or chronic pain. Future research should include patients with clinical pain to explore the discrepancy in brain mechanisms between healthy and clinical pain individuals.

In summary, the current study reported that the impairment of perception contributed to a fear overgeneralization in pain conditions, which led to increasing vigilance and reduced attention to the pain‐related stimulus. The present findings offer insights into psychobiological processes involved in fear conditioning and the generalization of an acute painful state, which may provide new evidence for the role of pain‐related fear in the transition from acute to chronic pain.

## AUTHOR CONTRIBUTIONS

**Xiaomin Huang**: Conceptualization; methodology; software; formal analysis; investigation; writing—original draft. **Jiali Chen and Xianglong Wang**: Methodology; formal analysis; investigation; writing—original draft. **Xuefei Zhang and Junqin Ma**: Methodology; software; data curation. **Sishi Liu and Xinli Liu**: Methodology; software; visualization. **Qiling Ou and Wenwei Tan**: Resources; writing—review & editing. **Wen Wu**: Conceptualization; methodology; supervision; project administration; funding acquisition; writing—original draft.

### PEER REVIEW

The peer review history for this article is available at https://publons.com/publon/10.1002/brb3.3050.

## Data Availability

I confirm that my article contains a Data Availability Statement even if no data are available.
